# Variations in the length of stay of intensive care unit nonsurvivors in three scandinavian countries

**DOI:** 10.1186/cc9279

**Published:** 2010-10-04

**Authors:** Kristian Strand, Sten M Walther, Matti Reinikainen, Tero Ala-Kokko, Thomas Nolin, Jan Martner, Petteri Mussalo, Eldar Søreide, Hans K Flaatten

**Affiliations:** 1 Health Services Research Centre, Akershus University Hospital, Sykehusveien 25, 1478 Lørenskog, Norway; 2 Department of Anaesthesia and Intensive Care, Stavanger University Hospital, Armauer Hansens vei 20, 4068 Stavanger, Norway; 3 Department of Cardiothoracic Anaesthesia and Intensive Care, Linkøping University Hospital, 581 85 Linkøping, Sweden; 4 Department of Intensive Care, North Karelia Central Hospital, Tikkamaentie 16, 80210 Joensuu, Finland; 5 Department of Anesthesiology, Division of Intensive Care, Oulu University Hospital, P.O. Box 21, 90029 OUH, Oulu, Finland; 6 Department of Anaesthesia and Intensive Care, Kristianstad Hospital, 291 85 Kristianstad, Sweden; 7 Department of Anaesthesia, Sahlgrenska University Hospital/Molndal, 431 80 Molndal, Sweden; 8 Tieto Healthcare and Welfare, P.O. Box 1188, FI-70211 Kuopio, Finland; 9 Department of Anesthesia and Intensive Care, Haukeland University Hospital, Jonas Liesvei 65, 5021 Bergen, Norway; 10 Department of Surgical Sciences, University of Bergen, Jonas Liesvei 65, 5020 Bergen, Norway

## Abstract

**Introduction:**

The length of stay (LOS) in intensive care unit (ICU) nonsurvivors is not often reported, but represents an important indicator of the use of resources. LOS in ICU nonsurvivors may also be a marker of cultural and organizational differences between units. In this study based on the national intensive care registries in Finland, Sweden, and Norway, we aimed to report intensive care mortality and to document resource use as measured by LOS in ICU nonsurvivors.

**Methods:**

Registry data from 53,305 ICU patients in 2006 were merged into a single database. ICU nonsurvivors were analyzed with regard to LOS within subgroups by univariate and multivariate analysis (Cox proportional hazards regression).

**Results:**

Vital status at ICU discharge was available for 52,255 patients. Overall ICU mortality was 9.1%. Median LOS of the nonsurvivors was 1.3 days in Finland and Sweden, and 1.9 days in Norway. The shortest LOS of the nonsurvivors was found in patients older than 80 years, emergency medical admissions, and the patients with the highest severity of illness. Multivariate analysis confirmed the longer LOS in Norway when corrected for age group, admission category, sex, and type of hospital. LOS in nonsurvivors was found to be inversely related to the severity of illness, as measured by APACHE II and SAPS II.

**Conclusions:**

Despite cultural, religious, and educational similarities, significant variations occur in the LOS of ICU nonsurvivors among Finland, Norway, and Sweden. Overall, ICU mortality is low in the Scandinavian countries.

## Introduction

Mortality and length of stay (LOS) are two frequently reported outcomes in intensive care units (ICUs). Vital status at ICU discharge is easily obtained in most units, but often, a more-robust outcome measure such as hospital mortality or mortality at a specific time point is preferred, because they are less likely to be influenced by organizational factors. Nevertheless, ICU mortality still plays a large part in ICU audits, as it may be combined with the LOS and hospital mortality to monitor resource utilization.

A specific group of patients that may be characterized by the combination of these measures is the patients who die during their ICU stay. Resource use in these patients, as measured by LOS in the ICU, may be sensitive to organizational and cultural differences between units, such as the availability of high-dependency units and variations in end-of-life practices between different countries. However, not many studies have focused specifically on LOS in ICU nonsurvivors and its relation to various geographic and organizational factors.

The three neighboring countries (Finland, Norway, and Sweden) share close historic and cultural ties that have resulted in several common traits. ICUs in the Scandinavian countries are run predominantly by anesthesiologists. The clinical training in intensive care is organized by the Scandinavian Society of Anaesthesiology and Intensive Care (SSAI) with a 2-year training program in intensive care medicine established in 1999 [1]. It is believed that the similarities in the organization and practice of intensive care medicine in the Scandinavian countries have led to similar case-mixes and outcomes. All three countries have national intensive care registries that cover a majority of ICU admissions [2-4]. To compare and report national intensive care data from these three countries, we created a merged database of all registered admissions in 2006.

The primary aim of this study was to report intensive care mortality and to document resource use as measured by LOS in ICU nonsurvivors by using a merged database of 53,305 ICU admissions in Norway, Finland, and Sweden in 2006. We analyzed the significance of several variables with regard to LOS to identify national and organizational differences in the treatment of ICU nonsurvivors.

## Materials and methods

**Figure 1:**
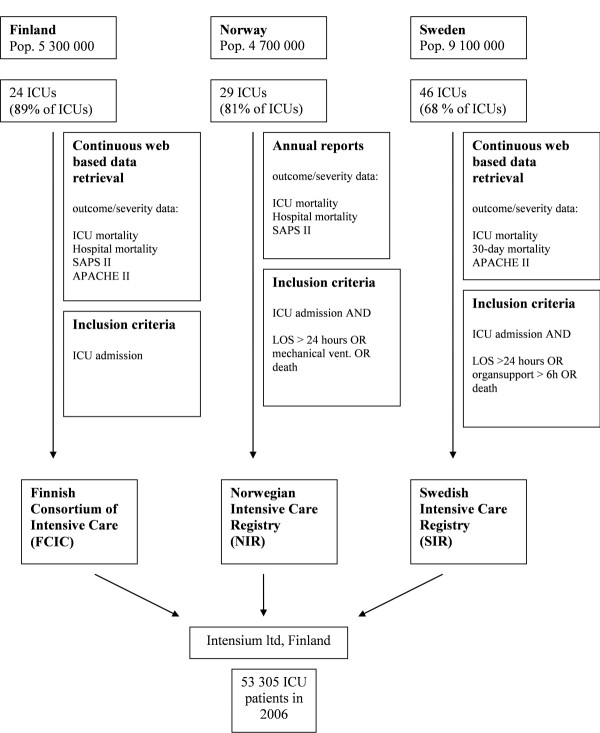
**The 2006 Scandinavian ICU cohort collection of data**.

The dataset was compiled by a collaboration of the national intensive care registries of Finland, Norway, and Sweden. Data from all ICU admissions in 2006 were merged into one database by Intensium Ltd., Finland [3], and resulted in a database of 53,305 patients. Data collection is illustrated in Figure 1.

The national registries of Finland, Norway, and Sweden are different with regard to organization, collected variables, and modes of data collection. The most important difference is linked to the definition of ICU patients when LOS is shorter than 24 hours. Most small nonuniversity hospitals in Norway and Sweden have combined postoperative and intensive care units. In these combined units, postoperative patients with LOS longer than 24 hours are defined as ICU patients. In Norway, all patients who receive mechanical ventilation during their ICU stay are defined as ICU patients. The Swedish registry includes postoperative admissions if organ support beyond normal postoperative recovery is required (> 6 hours). The Finnish registry collects data for all ICU admissions. Patients who die during their ICU stay are defined as ICU patients in all three registries, regardless of LOS. To adjust for differences in registration thresholds for the whole cohort, we performed additional mortality analysis for patients with LOS longer than 24 hours.

Automatic data retrieval by clinical information systems was used in 15 of 24 Finnish ICUs in 2006. The Norwegian and Swedish registries did not receive data based on automated systems. LOS was calculated as the number of hours spent in the ICU converted to days and fractions of days in all registries.

The steering committees of all three registries approved the project. The regional ethics committee (Western Norway Regional Health Authority, Norway) waived approval because the project involved routinely collected, anonymous data from governmentally approved quality registries.

### Statistics

LOS is presented as medians and quartiles (IQR) unless otherwise stated, as the distribution is highly skewed. Other continuous variables are presented as means and standard deviations (SDs). Analyses of LOS were done with the Kruskal-Wallis, log-rank or Mann-Whitney *U *test, where appropriate. For continuous variables, the means were analyzed with the Student *t *test or one-way analysis of variance, where appropriate. Categoric variables were analyzed by using the χ^2 ^test. APACHE II and SAPS II, both without age points, were grouped into quartiles before univariate analysis. To examine the independent effect of several variables on LOS, we performed a multivariate Cox regression proportional-hazards analysis, which included age category, admission category, hospital type, country, and gender. The proportional-hazards assumption was assessed graphically with relevant covariates. We used SPSS version 15.0 (SPSS Inc., Chicago, IL).

## Results

Vital status at ICU discharge was available for 53,255 patients. Overall, 4,854 patients (9.1%) died during the ICU stay (Table 1). The median time to death in the ICU was 1.5; IQR, 0.5 to 4.2 (mean, 4.3 ± 9.1) days (Table 2). Overall LOS was 1.6; IQR, 0.9 to 3.6 days. Severity, as measured by APACHE II and SAPS II, was higher in Finland than in Norway or Sweden.

**Table 1:** Patient characteristics

	Finland	Norway	Sweden	Total
Number of patients, *n*	14,614	10,988	27,653	53,255
Male (%)	63.0	52.8	56.7	57.7
Age, (years) mean (SD)	58.0 (18.6)	58.9 (22.6)	55.1 (23.2)	56.7 (22.0)
ICU mortality (%)	8.6	12.4	8.1	9.1
Hospital mortality (%)	17.0	16.8	n.a.	16.9
30-day mortality (%)	n.a	n.a	16.6	16.6
LOS (days) median (IQR)	1.6 (0.9-3.6)	2.1 (1.2-4.9)	1.0 (0.5-2.2)	1.3 (0.7-3.1)
SAPS II, mean (SD)	38.2 (18.7)	36.6 (18.3)	n.a.	37.5 (18.5)
APACHE II, mean (SD)	20.4 (9.2)	n.a.	15.5 (8.8)	17.5 (9.3)

LOS, length of stay in the ICU, n.a., not available. Characteristics are not corrected for different registration thresholds in the three registries.

**Table 2:** ICU nonsurvivors

	Total	Finland	Norway	Sweden	***P***^**a**^
Number of deaths in ICU	4,853	1,257	1,358	2,238	--
Male (%)	57.3	62.9	53.8	46.2	< 0.001
Age (years), mean (SD)	67.2 (16.8)	64.1 (15.5)	68.0 (18.1)	68.5 (16.4)	< 0.001
LOS (days) median (IQR)	1.5 (0.5-4.2)	1.3 (0.5-3.8)	1.9 (0.6-5.4)	1.3 (0.5-3.6)	< 0.001
LOS (days) mean (SD)	4.3 (9.1)	3.7 (7.8)	5.5 (10.6)	4.0 (8.7)	--
SAPS II, mean (SD)	61.5 (19.4)	65.2 (19.9)	57.7 (18.1)	n.a.	< 0.001
APACHE II, mean (SD)	29.9 (9.0)	32.8 (9.6)	n.a.	27.8 (8.0)	< 0.001

LOS, length of stay in the ICU; n.a., not available. **^a^**Comparisons between countries: Age analyzed with one-way ANOVA; gender, with χ^2 ^test for categoric variables; LOS analyzed with Kruskal-Wallis; SAPS II and APACHE II analyzed with the Student *t *test.

Some 31, 727 patients had LOS longer than 24 hours. In this group, ICU mortality was 9.2%, and overall LOS was 3.3; IQR, 1.7 to 6.7 days (Table 3).

**Table 3:** Patients with LOS longer than 24 hours

	Finland	Norway	Sweden	Total
Number of patients, *n*	9,154	9,214	13,359	31,727
Male (%)	65.5	54.0	58.1	59.1
Age (years) mean (SD)	59.0 (17.6)	59.4 (22.2)	59.5 (20.8)	59.3 (20.4)
ICU mortality (%)	7.8	10.0	9.7	9.2
Hospital mortality (%)	18.4	14.3	n.a.	16.4
30-day mortality (%)	n.a.	n.a.	19.7	19.7
LOS (days) median (IQR)	3.3 (1.7-6.7)	4.0 (2.0-9.7)	3.1 (1.8-7.3)	3.3 (1.8-7.8)
SAPS II, mean (SD)	41.5 (17.4)	36.4 (17.3)	n.a.	39.1 (17.6)
APACHE II, mean (SD)	22.3 (8.6)	n.a.	17.8 (8.4)	17.5 (9.3)

LOS, length of stay in the ICU; n.a., not available.

**Figure 2:**
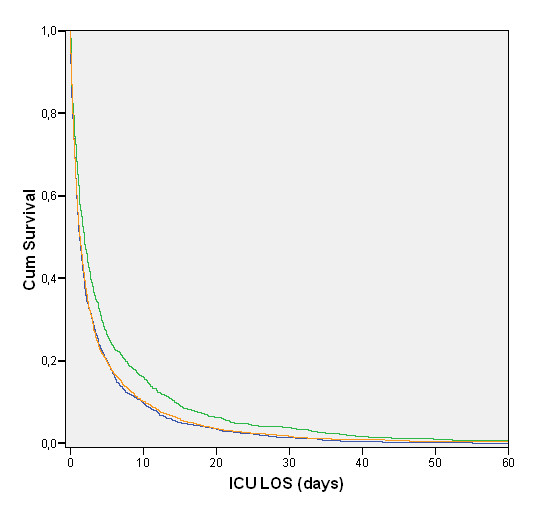
**Time to death after ICU admission**. Blue line, Finland; orange line, Sweden; green line, Norway.

The median time to death in Norway was 1.9; IQR, 0.6 to 5.4 days, which differed significantly from that in Finland: 1.3; IQR, 0.5 to 3.8 days, and Sweden: 1.3; IQR, 0.5 to 3.6 days (Figure 2). ICU nonsurvivors used 12.4% of the total number of ICU days. The shortest LOS of the nonsurvivors was found in patients older than 80 years, emergency medical admissions, nonuniversity hospital admissions, female patients, and the quartiles with the highest severity scores without age points (Table 4).

**Table 4:** LOS (days) of ICU nonsurvivors in subgroups

		Total	Finland	Norway	Sweden	***P***^**a**^
Age group (years)						
					
	0-40	1.5 (0.4-4.3)	1.3 (0.3-5.1)	1.9 (0.6-4.8)	1.3 (0.4-3.5)	0.17
	40-60	1.7 (0.7-4.6)	1.4 (0.6-3.7)	2.2 (0.8-7.1)	1.7 (0.7-5.0)	0.02
	60-80	1.7 (0.6-5.2)	1.4 (0.5-4.7)	2.5 (0.8-7.8)	1.6 (0.6-4.3)	< 0.001
	> 80	1.0 (0.3-2.6)	0.9 (0.2-2.0)	1.3 (0.4-3.1)	0.9 (0.3-2.3)	0.007
	*P***^a^**	< 0.001				
					
Admission category						
					
	Elective surgery	2.5 (1.1-7.4)	1.7 (0.8-5.3)	3.4 (1.2-8.5)	3.1 (1.1-7.2)	0.168
	Emergency medical	1.3 (0.4-3.8)	1.1 (0.4-3.5)	2.1 (0.7-5.9)	1.2 (0.4-3.4)	< 0.001
	Emergency surgical	1.9 (0.7-5.2)	1.9 (0.8-4.9)	2.0 (0.6-5.1)	1.9 (0.6-6.2)	0.961
	*P***^a^**	< 0.001				
					
Type of hospital						
					
	Nonuniversity	1.3 (0.4-4.0)	1.1 (0.4-3.9)	1.9 (0.5-5.3)	1.2 (0.4-3.4)	< 0.001
	University	1.6 (0.6-4.3)	1.5 (0.6-3.8)	2.2 (0.8-6.0)	1.7 (0.6-4.4)	< 0.001
	*P***^a^**	< 0.001				
					
Gender						
					
	Female	1.3 (0.5-3.9)	1.1 (0.4-3.4)	1.7 (0.6-5.1)	1.2 (0.4-3.4)	< 0.001
	Male	1.6 (0.5-4.5)	1.4 (0.5-4.0)	2.1 (0.6-5.6)	1.5 (0.5-3.9)	< 0.001
	*P***^a^**	0.001				
					
SAPS II quartiles^b^						
					
	1. (0-35)	3.7 (1.2-10.2)	3.7 (0.9-10.0)	3.6 (1.2-10.6)	n.a.	0.538
	2. (36-48)	2.3 (0.7-6.4)	2.2 (0.6-6.0)	2.3 (0.8-7.0)	n.a.	0.509
	3. (49-62)	1.4 (0.6-3.5)	1.2 (0.6-2.7)	1.8 (0.6-4.2)	n.a.	0.003
	4. (≥63)	0.9 (0.3-1.8)	0.8 (0.3-1.5)	1.1 (0.5-2.7)	n.a.	< 0.001
	*P***^a^**	< 0.001				
					
APACHE II quartiles^b^						
					
	1. (0-19)	2.7 (0.7-7.7)	3.3 (0.4-10.2)	n.a.	2.5 (0.8-7.2)	0.712
	2. (20-25)	2.0 (0.7-5.0)	2.2 (0.4-5.4)	n.a.	1.9 (0.8-4.4)	0.580
	3. (26-30)	1.2 (0.5-3.0)	1.4 (0.5-3.7)	n.a.	1.2 (0.5-2.5)	0.103
	4. (≥33)	0.9 (0.4-1.8)	0.9 (0.4-1.7)	n.a.	0.8 (0.4-1.9)	0.703
	*P***^a^**	< 0.001				

LOS, length of stay in the ICU; n.a., not available. **^a^**Kruskal-Wallis or Mann-Whitney *U *test, where appropriate.

^b^APACHEII/SAPS II points, age points deducted.

In the multivariate Cox regression analysis, the following variables were found to be independently associated with LOS: age group, country, admission category, and sex (Table 5). No significant association was found for the type of hospital.

**Table 5:** Multivariate analysis of the relation between selected variables and LOS in ICU nonsurvivors

		Number	HR (CI, 95%)	*P*
Age group (years)				
			
	0-40	282	1.00 Reference	
	40-60	901	0.93 (0.82-1.07)	0.317
	60-80	2,320	0.94 (0.83-1.07)	0.325
	> 80	1,157	1.46 (1.28-1.67)	< 0.001
			
Admission category				
			
	Emergency surgical	982	1.00 Reference	
	Elective surgical	123	0.91 (0.75-1.10)	0.318
	Emergency medical	3,555	1.23 (1.14-1.32)	< 0.001
			
Country				
			
	Finland	1,254	1.00 Reference	
	Norway	1,172	0.74 (0.68-0.80)	< 0.001
	Sweden	2,234	0.92 (0.86-0.99)	0.156
			
Sex				
			
	Male	2,679	1.00 Reference	
	Female	1,981	1.10 (1.03-1.16)	0.003
			
Type of hospital				
			
	Nonuniversity	3,082	1.00 Reference	
	University	1,578	1.03 (0.97-1.10)	0.301

LOS, length of stay in ICU; HR, hazard ratio (higher HR means shorter LOS).

**Figure 3:**
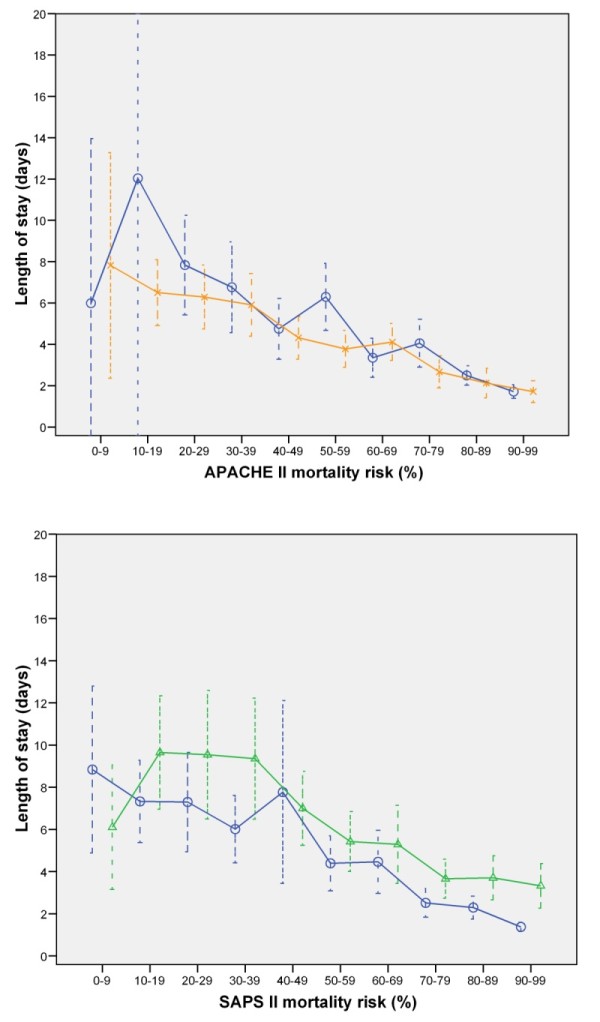
**Predicted mortality and length of stay in nonsurvivors**. Mean length of stay and 95% confidence intervals in relation to predicted mortality by APACHE II (Finland, Sweden) and SAPS II (Finland, Norway). Circle/blue line, Finland; x/orange line, Sweden; triangle/green line, Norway.

The maximal LOS of ICU nonsurvivors was found in patients with a predicted mortality of 10% to 20% by using SAPS II and APACHE II (Figure 3).

## Discussion

In this study of a large number of ICU admissions from 2006 in Finland, Norway, and Sweden, the ICU mortality was found to be low (9.1%). Only a few studies in intensive care have reported ICU mortality on a national level. In a study from Australia and New Zealand, the bi-national registry reported an ICU mortality of 9% for 2003 [5], whereas the Italian national registry (GiViTi) reported an ICU mortality of 16.9% for 2005 [6]. Multinational studies have reported ICU mortality to range from 7% to 20%. Although such multinational studies often provide greater detail than national registries with regard to the data of individual patients, their ability to characterize national outcomes is limited because the representativeness of the participating units may be questioned. This is illustrated by the SAPS 3 study [7], in which the Northern European region was represented by only 355 patients with an ICU mortality of 20%, which is obviously not representative for our three countries. The reasons for the low mortality in the Scandinavian countries remain to be established, but because ICU-bed availability in Finland, Norway, and Sweden is low (approximately five to six per 100,000 population), and severity of illness is high, regional prevalence of diseases, socioeconomic factors, and health care quality are more likely explanations. A recent study of critical care systems across North America and Europe reported a negative correlation between the number of ICU beds and hospital mortality [8]. The number of ICU beds in Europe varied from 3.5 (UK) to 24.6 per 100,000 population (Germany). When compared with the countries with similar ICU-bed availability in that study, mortality in our study was low.

Because ICU mortality is influenced by organizational factors in hospitals and health systems, hospital mortality is generally regarded as a better outcome measure. The main problem of using hospital mortality when comparing outcomes is bias due to interhospital transfers. In health systems in which such transfers are common and no routine registration of vital status after transfer is present, such comparisons will be biased in favor of hospitals transferring the highest number of patients [9]. A low ICU mortality coupled with high hospital mortality could be a marker of premature discharges from the ICU or poor post-ICU care. However, our data suggest that the hospital (Finland, Norway) or 30-day mortality (Sweden) of ICU patients is low in the Scandinavian countries, but the lack of standardized outcome measures, uncertainties regarding transfer follow-up, and different registration thresholds of patients with short LOS make exact comparisons within the Scandinavian countries inaccurate.

Measuring the use of resources in individual ICU patients is not a straightforward procedure. Several nursing-activity scores have been developed, and their use does provide important information not obtained when using the crude LOS [10,11]. Our registries gather data on nursing activity, but the use of different scoring systems precludes comparisons between our countries. We have therefore used the LOS in our analysis, which is the main determinant of resource use and is readily available in most studies.

LOS is also influenced by severity of illness, and several studies have attempted to create severity-based LOS-prediction models [12,13]. The LOS of nonsurvivors has been difficult to model, as the relation between LOS and severity differs from that of the general ICU population. In contrast to ICU survivors, who have increasing LOS with increasing severity at ICU admission, an inverse relation is found between severity and LOS in ICU nonsurvivors (Figure 3). We found the longest LOS in the group of 10% to 20% mortality risk, which is in accordance with an earlier study by the Scottish national ICU registry [14]. This means that the short LOS in the Finnish nonsurvivors may in part be explained by the higher severity of these patients' illness. Treatment limitations in the very old may have influenced our findings, but the shorter LOS in the groups with higher severity scores was present even after removing age points.

In the process of admitting a patient to the ICU, a need exists for medical, prognostic, and ethical considerations to admit patients who are likely to benefit from treatment in the ICU. Limiting the use of resources in patients who ultimately will not benefit from intensive care is essential, as the availability of ICU beds is limited in most hospitals. In the ETHICUS study, the Northern European region was shown to have the highest prevalence of withholding/withdrawal of therapy in Europe [15]. Protestant or nonreligious doctors, representing the most common religious views in the Scandinavian countries, instituted treatment limitations earlier after ICU admittance than did doctors with other religious affiliations [16]. The Italian GiViTi group reported a median LOS of 3.0 days (mean LOS of 8.4 days) in ICU nonsurvivors during 2005 [6]. Compared with the Italian LOS, the median LOS of 1.5 days among Scandinavian ICU nonsurvivors is remarkably low, but may in part be explained by differences in culture and religion. It should be noted that GiViTi does not use the Exact method for calculating LOS, and this may account for some of the difference between Italy and Scandinavia.

Among the three countries, we found a longer LOS in the Norwegian patients who died during their ICU stay compared with the patients in the neighboring countries. This difference was highly statistically significant, even after corrections for other factors through Cox regression analysis, but the proportion of variation explained by this model is not easily determined. Severity of illness was not included in the model and may explain some of the differences. When we included SAPS II without age points in a separate multivariate analysis of only Finland and Norway, both the levels of severity and nationality were highly significant. It should be noted that the higher severity scores in the Finnish patients may in part be due to a more frequent use of automatic data retrieval, which has been shown to increase scores through higher sampling rates [17].

The reasons for the differences in LOS in nonsurvivors among the three countries are not apparent. One explanation might be differences in the discharge practice of these patients, as indicated by the lower ICU mortality and higher post-ICU mortality in Finland and Sweden compared with Norway.

The increased LOS in Norway represents a prolonged stay of 14.4 hours per nonsurvivor, which is approximately 3.5% of total LOS in the Norwegian cohort. It is not obvious that an increase in LOS of this magnitude is of clinical relevance, but when ICU-bed availability is low, even small increases in LOS may have an impact on admission and discharge policies. The incidence of nighttime discharge could be a marker of ICU-bed shortage, but such data are not available for all three countries in the current database.

Conflicting data are found on the influence of old age on ICU mortality [18,19], which is probably due to differences in admission policies and intensity of treatment. In our study ICU mortality in the patients aged 80 years or older was 16.9% which was higher than that in the other age groups (2.7% to 11.6%). The short LOS in nonsurvivors aged 80 years and older compared with nonsurvivors between 60 and 80 years is striking and may, in our opinion, represent adherence to the life-cycle principle in which rationing is performed on the basis of age as well as prognosis [20]. An earlier Finnish study of the ICU treatment of the elderly explained the short LOS in the very old to be caused by restrictions in therapy, but also by a greater number of early deaths [21].

Our study is based on a database of 53,503 patients, making it one of the largest studies on ICU patients in the Scandinavian countries. It is the first study to provide data from several national registries to compare directly the practice of intensive care medicine in these countries. Although the registries are believed to cover the vast majority of ICUs in all countries, inevitably, some ICUs are missing in the database because of the voluntary data submission. Data on hospital mortality in Sweden are not available, and direct comparison is hence not possible for the three countries. Another problem in the comparison and description of Scandinavian intensive care is the different thresholds for registering patients in the registries. Firm conclusions on the differences in mortality and LOS among the Scandinavian countries are not possible with these limitations in mind. However, analysis of LOS in nonsurvivors was not affected by registration differences because all registries registered all deaths in the ICU, regardless of LOS. Analysis of patients with LOS longer than 24 hours confirmed the low ICU mortality and differences in overall LOS among the three countries.

The variation in definitions and registered variables was a major obstacle when constructing the merged database used in this study and precludes us from pointing to any definitive causal relation for the differences in LOS. In our study, inclusion of a common measure of severity in the multivariate analysis would have been of particular interest. More-detailed information on case mix, organization, and treatment limitations will have great interest in future analyses. A European consensus on core variables and definitions in ICU registration would probably be a valuable step in the provision of such data.

## Conclusions

In this database of 53,305 ICU patients in Finland, Sweden, and Norway admitted during 2006, we found an ICU mortality of 9.1%, which is considered low compared with reports from other countries. ICU mortality was similar in the three countries. The median LOS of ICU nonsurvivors was only 1.5 days, but a markedly longer LOS was noted in Norway than in the other participating countries. This was confirmed in the multivariate analysis, in which the shortest LOS was found in patients aged older than 80 years and in emergency medical admissions.

## Key messages

• Length of stay of ICU nonsurvivors is seldom reported, but may give important information on organization, resource use, and cultural differences.

• Length of stay of ICU nonsurvivors is short in Scandinavia (1.5 days), but is longer in Norway than in Finland and Sweden.

• Old age and high severity of illness are associated with short LOS in ICU nonsurvivors.

• Overall ICU mortality in Scandinavia is low (9.1%).

## Abbreviations

ICU: Intensive Care Unit; LOS: length of stay in ICU.

## Competing interests

The authors declare that they have no competing interests.

## Authors' contributions

KS drafted the manuscript. KS, HKF, and SMW performed the statistical analyses. PM created the merged database. HKF conceived the study. All authors revised the manuscript for important intellectual content. All authors read and approved the final manuscript.

## References

[B1] Scandinavian Society of Anaesthesiology and Intensive Care Medicine http://www.ssai.info/Education/intensive_care.html

[B2] Swedish Intensive Care Registry http://www.icuregswe.org

[B3] Intensium Ltd http://www.intensium.com/web/english

[B4] Norwegian Intensive Care Registry http://www.intensivregister.no

[B5] Moran JL Bristow P Solomon PJ George C Hart GK Mortality and length-of-stay outcomes, 1993-2003, in the binational Australian and New Zealand intensive care adult patient database Crit Care Med 2008 36 46 61 10.1097/01.CCM.0000295313.08084.58 18090383

[B6] Boffelli S Rossi C Anghileri A Giardino M Carnevale L Messina M Neri M Langer M Bertolini G Marco A Arnaldo A Armando A Patrizia A Stefani AB Davide A Enrico A Flavio B Balata A Massimo B Remo B Arcangelo B Teresa B Francesco B Eduardo B Giuseppe BU Maurizio B Olga BM Andre B Angelo B Daniela BR Continuous quality improvement in intensive care medicine: the GiViTI Margherita project: report 2005 Minerva Anestesiol 2006 72 419 432 16682911

[B7] Metnitz PG Moreno RP Almeida E Jordan B Bauer P Campos RA Iapichino G Edbrooke D Capuzzo M Le Gall JR SAPS 3: from evaluation of the patient to evaluation of the intensive care unit, Part 1: objectives, methods and cohort description Intensive Care Med 2005 31 1336 1344 10.1007/s00134-005-2762-6 16132893 PMC1315314

[B8] Wunsch H Angus DC Harrison DA Collange O Fowler R Hoste EA de Keizer NF Kersten A Linde-Zwirble WT Sandiumenge A Rowan KM Variation in critical care services across North America and Western Europe Crit Care Med 2008 36 2787 2793 e2781-e2789 10.1097/CCM.0b013e318186aec8 18766102

[B9] Kahn JM Kramer AA Rubenfeld GD Transferring critically ill patients out of hospital improves the standardized mortality ratio: a simulation study Chest 2007 131 68 75 10.1378/chest.06-0741 17218558

[B10] Keene AR Cullen DJ Therapeutic intervention scoring system: update 1983 Crit Care Med 1983 11 1 3 10.1097/00003246-198301000-00001 6848305

[B11] Reis D Miranda Moreno R Iapichino G Nine equivalents of nursing manpower use score (NEMS) Intensive Care Med 1997 23 760 765 10.1007/s001340050406 9290990

[B12] Vasilevskis EE Kuzniewicz MW Cason BA Lane RK Dean ML Clay T Rennie DJ Vittinghoff E Dudley RA Mortality probability model III and simplified acute physiology score II: assessing their value in predicting length of stay and comparison to APACHE IV Chest 2009 136 89 101 10.1378/chest.08-2591 19363210 PMC3198495

[B13] Niskanen M Reinikainen M Pettila V Case-mix-adjusted length of stay and mortality in 23 Finnish ICUs Intensive Care Med 2009 35 1060 1067 10.1007/s00134-008-1377-0 19125233

[B14] Woods AW MacKirdy FN Livingston BM Norrie J Howie JC Evaluation of predicted and actual length of stay in 22 Scottish intensive care units using the APACHE III system: acute physiology and chronic health evaluation Anaesthesia 2000 55 1058 1065 10.1046/j.1365-2044.2000.01552.x 11069331

[B15] Sprung CL Cohen SL Sjokvist P Baras M Bulow HH Hovilehto S Ledoux D Lippert A Maia P Phelan D Schobersberger W Wennberg E Woodcock T End-of-life practices in European intensive care units: the Ethicus Study JAMA 2003 290 790 797 10.1001/jama.290.6.790 12915432

[B16] Sprung CL Maia P Bulow HH Ricou B Armaganidis A Baras M Wennberg E Reinhart K Cohen SL Fries DR Nakos G Thijs LG The importance of religious affiliation and culture on end-of-life decisions in European intensive care units Intensive Care Med 2007 33 1732 1739 10.1007/s00134-007-0693-0 17541550

[B17] Suistomaa M Kari A Ruokonen E Takala J Sampling rate causes bias in APACHE II and SAPS II scores Intensive Care Med 2000 26 1773 1778 10.1007/s001340000677 11271084

[B18] de Rooij SE Govers A Korevaar JC Abu-Hanna A Levi M de Jonge E Short-term and long-term mortality in very elderly patients admitted to an intensive care unit Intensive Care Med 2006 32 1039 1044 10.1007/s00134-006-0171-0 16791666

[B19] Bagshaw SM Webb SA Delaney A George C Pilcher D Hart GK Bellomo R Very old patients admitted to intensive care in Australia and New Zealand: a multi-centre cohort analysis Crit Care 2009 13 R45 10.1186/cc7768 19335921 PMC2689489

[B20] White DB Katz MH Luce JM Lo B Who should receive life support during a public health emergency? Using ethical principles to improve allocation decisions Ann Intern Med 2009 150 132 138 19153413 10.7326/0003-4819-150-2-200901200-00011 PMC2629638

[B21] Reinikainen M Uusaro A Niskanen M Ruokonen E Intensive care of the elderly in Finland Acta Anaesthesiol Scand 2007 51 522 529 10.1111/j.1399-6576.2007.01274.x 17430311

